# First Detection of Critical Carbapenemase Genes (NDM, OXA-48, VIM) in Avian *Campylobacter* spp. Isolates in Tunisia: A Zoonotic and Public Health Concern

**DOI:** 10.3390/antibiotics14121236

**Published:** 2025-12-08

**Authors:** Manel Gharbi, Mohammed Abdo Saghir Abbas, Chadlia Hamdi, Safa Hamrouni, Abderrazak Maaroufi

**Affiliations:** 1 Group of Bacteriology and Biotechnology Development, Laboratory of Epidemiology and Veterinary Microbiology, Institut Pasteur de Tunis, University of Tunis El Manar (UTM), Tunis 1002, Tunisia; 2 Higher Institute of Biotechnology of Beja, University of Jendouba, Beja 9000, Tunisia; 3 Unit of Vector Ecology, Pasteur Institute of Tunis, Tunis 1002, Tunisia; mohammed.abbas@pasteur.utm.tn

**Keywords:** carbapenem-resistance, *Campylobacter* spp., poultry, *bla*_VIM_, *bla*_NDM_, *bla*_OXA-48_

## Abstract

**Background/Objectives**: The global emergence of carbapenem resistance is a major public health concern. *Campylobacter jejuni* and *Campylobacter coli*, key zoonotic agents causing human campylobacteriosis, are mainly isolated from poultry, their primary host. Their increasing resistance in animals and humans highlights the risk of gene transfer. This study investigates the molecular mechanisms of carbapenem resistance in 287 avian *Campylobacter* spp. isolates from Tunisia within a One Health approach. **Methods:** Antibiotic susceptibility of 287 carbapenem-resistant isolates, including 147 *C. jejuni* and 140 *C. coli*, was determined according to CLSI. All isolates were screened by PCR for genes encoding the most reported carbapenemases, including VIM, IMP, NDM and OXA-48. Eleven multidrug-resistant (MDR)/carbapenem-resistant *C. coli* isolates were selected to determine their clonal lineage by Multilocus sequence typing (MLST). **Results:** All isolates were susceptible to imipenem, but resistance to meropenem and ertapenem were observed in 60.71% and 35.71% of *C. coli* isolates, respectively, versus 13.6% in *C. jejuni* for each antibiotic. The *bla*_VIM_, *bla*_NDM_ and *bla*_OXA-48_ genes were detected in 15, 8, and 19 of the 20 *C. jejuni* isolates, respectively. However, for *C. coli*, 53, 12, and 15 isolates harbored *bla*_VIM_, *bla*_NDM_ and *bla*_OXA-48_ genes, respectively. The eleven (MDR)/carbapenem-resistant *C. coli* isolates belonged to a unique ST sequence type ST13450. **Conclusions:** We report for the first time the emergence of *bla*_VIM_, *bla*_NDM_, and *bla*_OXA-48_ genes in *Campylobacter* spp. isolates of poultry origin highlighting possible horizontal transfer of these genes to pathogenic Gram-negative bacteria of the poultry’s microbiota.

## 1. Introduction

Antibiotic resistance (AR) has emerged as a major public health threat over the past two decades [1,2]. The spread of AR is driven by both antibiotic-resistant bacteria (ARB) and mobile genetic elements carrying antibiotic resistance genes (ARGs) [3,4]. The overuse and misuse of antibiotics in human medicine and livestock production are key factors selecting for and disseminating ARB and ARGs, not only in clinical and agricultural settings but also in wildlife and environmental compartments such as soil and water [5]. Commensal bacteria colonizing livestock have been shown to act as important reservoirs of ARB and ARGs, which can spread to humans through direct contact, the food chain, or the surrounding environment [6]. These bacteria include zoonotic Enterobacterales (e.g., *Escherichia coli*, *Salmonella* spp.), *Staphylococcus* spp. (e.g., *Staphylococcus aureus*), *Enterococcus* spp., and *Campylobacter* spp. [6,7].

*Campylobacter* species are a leading cause of neonatal enteric disease in developing countries and a major contributor to foodborne diarrheal illness worldwide. Campylobacteriosis is an important zoonosis, with infection primarily acquired through the consumption of contaminated food, particularly poultry, or water, as well as through direct contact with infected food-producing animals or their carcasses [8]. More than 90% of human Campylobacter infections are attributed to *C. jejuni* and *C. coli.* Campylobacteriosis is typically a self-limiting illness characterized by diarrhea, fever, and abdominal pain. However, in severe cases, *Campylobacter* infections can lead to serious complications such as bloodstream infections (BSI), reactive arthritis, and Guillain–Barré syndrome (GBS) [9]. For severe infections, fluoroquinolones (e.g., ciprofloxacin) and macrolides (erythromycin, azithromycin) are the most commonly prescribed treatments for human campylobacteriosis; additionally, tetracycline and gentamicin remain effective options for systemic *Campylobacter* infections [10]. However, due to the excessive use of antibiotics in livestock, particularly in the poultry industry, high rates of antibiotic resistance have been reported in *Campylobacter* isolates [10,11]. This escalation has contributed to a growing pool of multidrug-resistant (MDR) isolates, particularly among strains associated with poultry production and contaminated food sources. The emergence of MDR *Campylobacter* poses a growing public health challenge, as it limits treatment options and increases the risk of severe or persistent infections [10,11].

Carbapenems (imipenem, meropenem, ertapenem) are considered a valuable antimicrobial class for managing complicated cases of campylobacteriosis that do not respond to first-line therapy [12]. Although carbapenem resistance in *Campylobacter* has historically been considered rare, recent reports from Europe and Asia indicate the emergence of carbapenem-resistant *Campylobacter* species, most frequently *C. coli*, often linked to prolonged selective antibiotic pressure, even though the underlying molecular mechanisms remain poorly defined [13,14]. Clinical and experimental studies have increasingly documented carbapenem non-susceptible *C. coli* and *C. jejuni* strains, particularly in immunocompromised patients and in poultry-associated isolates [14,15]. Evidence from several investigations shows that carbapenem non-susceptibility can develop in vivo following extended meropenem or ertapenem therapy, associated with mutations in *porA* and overexpression of β-lactamases such as *bla*_OXA-489_, pointing to adaptive resistance mechanisms under antibiotic pressure [16]. Recent systematic reviews on antibiotic resistance in *Campylobacter* species from humans, animals, and water sources in South Africa reported moderate levels of resistance to carbapenems. In human isolates, resistance rates reached 15.3% for imipenem and 19.3% for meropenem [17]. Similar trends were observed in isolates from meat products (imipenem: 23%), cattle (imipenem: 21.47%), and drinking water (meropenem: 15.0%). In Europe, surveillance data from 2021 to 2022 revealed notably high rates of ertapenem resistance in *C. coli* from animal sources: 42.1% in broilers (2022), 58.1% in fattening turkeys (2022), and 29.1% in cattle under one year of age (2021), while resistance remained low in fattening pigs (1.1% in 2021). In contrast, *C. jejuni* showed much lower ertapenem resistance, with rates of 9.2%, 15.1%, 0.0%, and 1.2% in broilers, fattening turkeys, cattle under one year, and fattening pigs, respectively [18]. Taken together, although the global prevalence of carbapenem-resistant *Campylobacter* remains low, this phenomenon is concerning and underscore the need for reinforced surveillance in both clinical and food-chain settings. Several molecular mechanisms contributing to multidrug resistance in *Campylobacter* isolates have been described, including active efflux pumps, chromosomal mutations, and enzymatic antibiotic modification [11,15]. Of particular concern are the acquired resistance mechanisms involving antibiotics considered critical for treating infections caused by other major pathogens such as Enterobacterales, enterococci, and staphylococci. These include carbapenem and linezolid resistance, which position *Campylobacter* species as potential reservoirs and vectors of mobile genetic elements within the intestinal microbiota, affecting both commensal and pathogenic bacteria [19,20,21].

The present study aimed to determine the prevalence of carbapenem resistance among previously characterized *C. jejuni* and *C. coli* isolates recovered from poultry feces and environmental samples, and to identify the molecular mechanisms associated with this resistance. Additionally, clonal relatedness among carbapenem-resistant isolates was assessed using multilocus sequence typing (MLST) in eleven multidrug-resistant *C. coli* isolates.

## 2. Results

### 2.1. Antimicrobial Susceptibility

**Figure 1:**
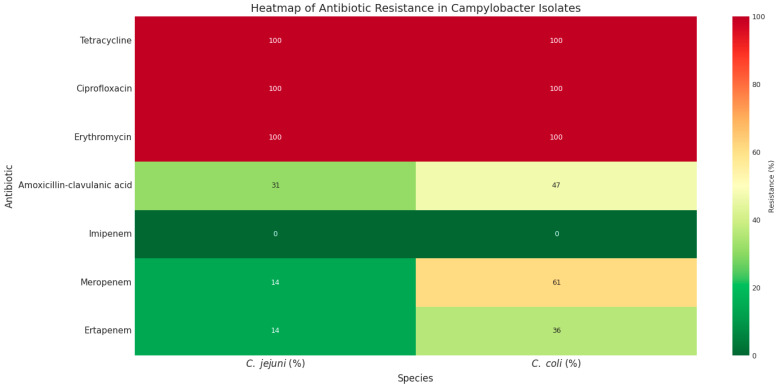
The heatmap of phenotypic antibiotic resistance patterns of *C. jejuni* and *C. coli* isolates. Green to red colors represent low to high resistance rates.

High rates of antibiotic resistance were observed among the 147 *C. jejuni* and 140 *C. coli* isolates (Table 1, Figure 1). All isolates were resistant to tetracycline, ciprofloxacin, and erythromycin. Resistance to amoxicillin–clavulanic acid was detected in 47.14% of *C. coli* and 31.19% of *C. jejuni* isolates.

Regarding carbapenem antibiotics, all isolates were susceptible to imipenem; however, resistance to meropenem and ertapenem was observed in 60.71% (*n* = 84) and 35.71% (*n* = 50) of *C. coli* isolates, respectively, compared with 13.6% (*n* = 20) of *C. jejuni* isolates for each antibiotic (Table 1). Notably, all 20 *C. jejuni* isolates were co-resistant to both meropenem and ertapenem. In contrast, among *C. coli* isolates, 15 (10.71%) were resistant only to ertapenem, 32 (22.85%) only to meropenem, and 93 (66.42%) were resistant to both ertapenem and meropenem (Figure 1).

### 2.2. Genes Encoding Carbapenem Resistance and Other Resistance Genes

**Figure 2:**
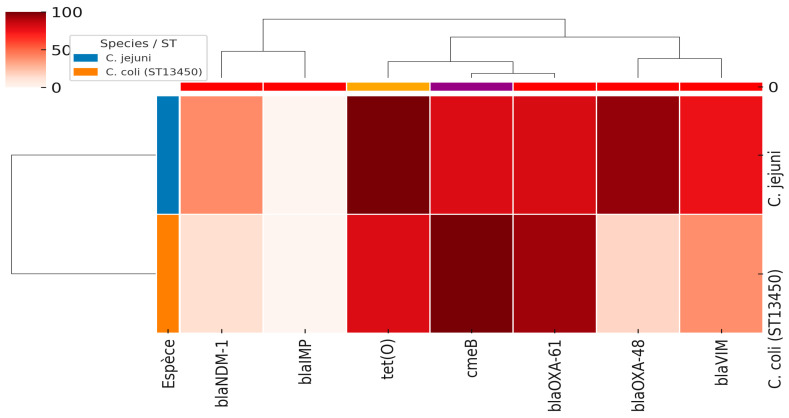
The heatmap of antimicrobial resistance genes in *C. jejuni* and *C. coli* (ST13450) isolates. The intensity of colors corresponds to the percentages of the detected genes (from 0 to 100%).

Among the four investigated genes encoding carbapenem resistance, the *bla*_VIM_, *bla*_NDM-1_, and *bla*_OXA-48_ genes were detected in 15/20, 8/20, and 19/20 of carbapenem-resistant *C. jejuni* isolates, respectively. However, for *C. coli*, 53, 12, and 15 isolates harbored *bla*_VIM_, *bla*_NDM_ and *bla*_OXA-48_ genes, respectively (Table 1, Figure 2). Notably, *bla*_IMP_ was absent in both species. Taken together, among the two species, *bla*_VIM_, *bla*_NDM-1_, and *bla*_OXA-48_ genes were detected in 68 (23.6%), 20 (6.9%), and 34 (13.9%) isolates, respectively.

**Figure 3:**
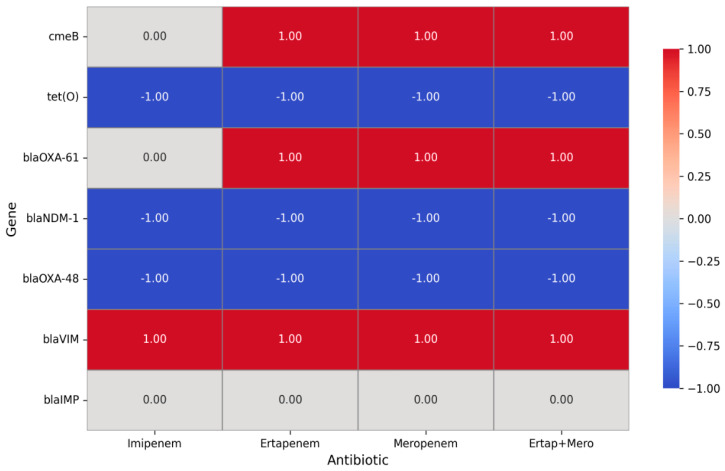
Correlation between carbapenem phenotypic resistance and carbapenemase resistance genes of *Campylobacter* isolates. A value of **1** represents a positive association, indicating that the presence of the gene is linked to increased resistance to the corresponding antibiotic. A value of **−1** denotes a negative association, meaning that the gene is associated with increased sensitivity. A value of **0** reflects a neutral or inconclusive effect, suggesting that no clear relationship between the gene and the antibiotic was identified.

We performed chi-square and Fisher’s exact tests to evaluate the association between carbapenemase genes and carbapenem resistance phenotypes in *C. jejuni* and *C. coli* isolates. The presence of *bla*_VIM_, *bla*_NDM_, and *bla*_OXA-48_ genes was significantly associated with carbapenem resistance (*p* < 0.01). Furthermore, a logistic regression model including these three genes confirmed that *bla*_VIM_ (OR = 16.5, *p* < 0.001), *bla*_OXA-48_ (OR = 6.7, *p* = 0.003), and *bla*_NDM_ (OR = 4.0, *p* = 0.012) independently contribute to resistance (Figure 3). These results support a strong genotypic–phenotypic correlation, strengthening the causal link between carbapenemase genes and resistance phenotypes in *Campylobacter* spp. isolates.

As investigated in our previous reports, Table 1 and Figure 2 illustrate the prevalence of major antimicrobial resistance genes among *C. jejuni* and *C. coli* isolates. In *C. jejuni*, *tet(O)* (100%) (Tetracycline resistance) and *bla*_OXA-61_ (81%) (bata-lactam resistance) were the most frequent resistance determinants, followed by *cmeB* (multi-drug efflux pump) (80%). In contrast, *C. coli* isolates exhibited 100% carriage of *cmeB*, together with high frequencies of *bla*_OXA-61_ (93%) and *tet(O)* (80%). These findings suggest that while both species share a high burden of efflux pump- and tetracycline-related resistance, there are interspecies differences in the distribution of β-lactamase and carbapenemase genes, which may reflect distinct evolutionary trajectories and selective pressures.

### 2.3. Clonality of Eleven C. coli Isolates

**Figure 4:**
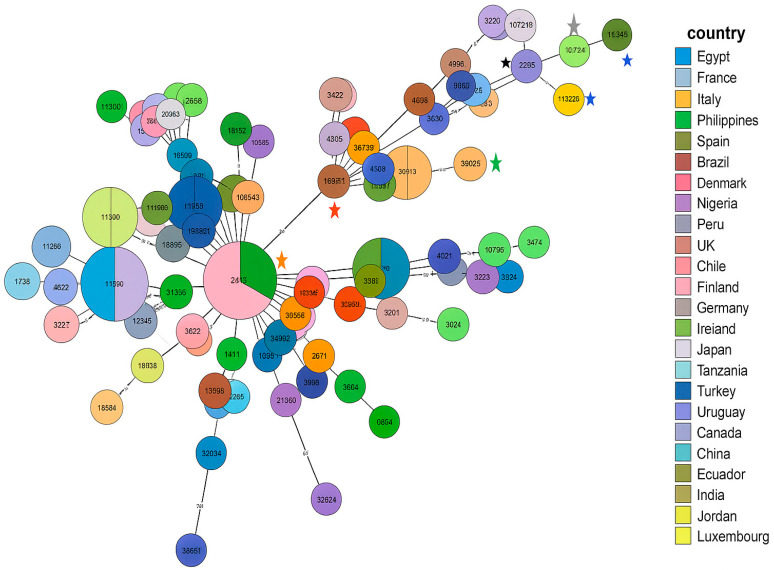
MLST analysis of the MDR *C. coli* ST13450 isolates compared with international MDR strains. Blue asterisk (ID118526): our strain; black asterisk: *C. coli* npCAMYO (ST8168); gray asterisk: *C. coli* Campy469 (ST7718); green asterisk: *C. coli* J19 (ST11913); red asterisk: *C. coli* FR1p365B (ST832); water green asterisk: *C. coli* FSIS32309315 (ST7818); orange asterisk: strains from Spain and Ireland belonging to ST827 (CC828).

All isolates belonged to the same sequence type, ST13450, which represents a newly identified ST. The eleven selected isolates reflect the different profiles of our multidrug-resistant *C. coli* isolates and were compared with other international multidrug-resistant strains, first according to the country of origin and then according to the type of disease, as shown in Figure 4. Each node represents a sequence type (ST), with node size proportional to the number of isolates. Colors indicate the country of origin.

The ST13450 clone detected in our eleven meropenem/ertapenem-resistant *C. coli* isolates is genetically related to strains from multiple regions worldwide (Europe, North Africa, South America, Asia), illustrating their integration within a global clonal network. The most closely related strain (ID: 80390) belongs to the ST8168 (CC828), presented by a strain (named as *C. coli* npCAMYO) from Peru, isolated in 2012 from human stool (carrier) (https://pubmlst.org/bigsdb?db=pubmlst_campylobacter_isolates&page=query, accessed on 25 November 2025). It was also closely related to an environmental Brazilian *C. coli* isolate (ID:32745, named Campy469) isolated in 2004 and belonging to the singleton ST7718. Additional examples illustrating the diversity of origins and isolation times within the cluster containing the Tunisian-related isolates include *C. coli* J19 (ID:112241), isolated in 2011 from chicken offal or meat in the Philippines and classified as the singleton ST11913; and *C. coli* FSIS32309315 (ID:143078), isolated in 2023 from chicken in the USA and assigned to ST7818 (CC828).

As shown in Figure 4, the first strain in this cluster is *C. coli* FR1p365B (ID:114327), isolated in 2022 from chicken offal or meat in Chile and assigned to ST832 (CC828). This strain, along with the rest of the cluster, appears to have evolved from a lineage predominantly composed of strains from Ireland and Spain, most of which belong to ST827 (CC828), indicating a close genetic relationship and a potential shared evolutionary origin.

**Figure 5:**
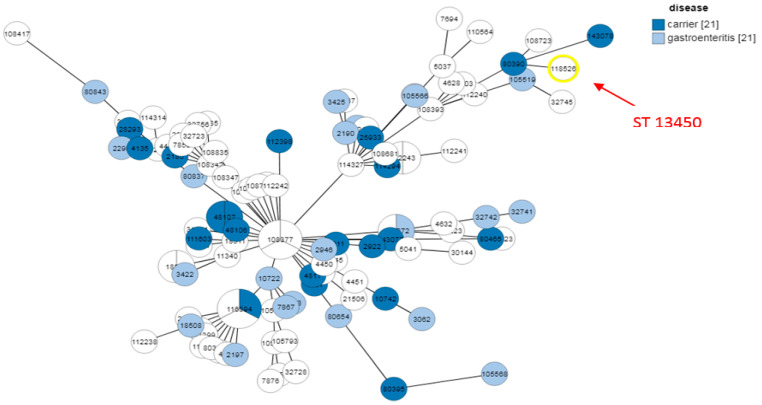
MLST analysis of ST13450 isolates according to clinical outcome and sources. The number 21 displayed next to ‘carrier’ and ‘gastroenteritis’ indicates the total number of strains included in each category, which were compared to our strain.

The disease-based MLST analysis in Figure 5 reveals that ST13450 isolates are found in both gastroenteritis cases and asymptomatic carriers. This dual distribution suggests that the clone has a pathogenic potential capable of causing clinical disease, while also persisting in a carrier state, which may facilitate its silent spread in human populations. Furthermore, the detection of related isolates in both animal sources (particularly chicken and chicken meat) and human samples supports the hypothesis of a zoonotic transmission cycle between animals and humans. These findings highlight the emerging and versatile nature of ST13450, demonstrating its ability to adapt to different hosts and clinical contexts. Therefore, this sequence type should be considered a potential zoonotic pathogen of public health concern.

## 3. Discussion

Globally, antibiotic resistance has emerged as important problem for human and animal health. According to the One Health approach, the dissemination of antibiotic-resistant bacteria (ARB), as well as genes encoding antibiotic resistance (ARGs), occurs in the human/animal/environment interface. In addition, several studies have reported the intra-and interspecies spread of mobile genetic elements encoding ARGs, mainly plasmids, transposons, and integrons, among isolates belonging to the same species and genera as well as between different bacterial families. This phenomenon is specifically enhanced in environments containing multiple phyla of bacteria such as the animal and human microbiome of gastrointestinal tract as well as aquatic environments and polluted soils [5,22].

Recent studies highlight the widespread prevalence of *Campylobacter* spp. in poultry and their environments, with contamination rates up to 36.7% in certain meat samples and frequent multidrug resistance, particularly to ciprofloxacin and tetracycline, though erythromycin remains largely effective. Intensive poultry farming contributes significantly to environmental pollution and the dissemination of antibiotics, pathogens, and antimicrobial resistance through manure, aerosols, and contaminated water, posing risks to both farm workers and surrounding communities [23]. Farm-level factors, especially drinking water supply systems and rural suppliers, were identified as significant risk factors for *Campylobacter* colonization in broiler flocks, underscoring the importance of biosecurity and environmental management to control pathogen spread [24].

Carbapenem resistance in Gram-negative bacteria has become a global concern. Enterobacteriaceae, *Pseudomonas aeruginosa*, and *Acinetobacter baumannii* resistant to carbapenems are classified as “critical” priority pathogens in the 2017 World Health Organization (WHO) global priority list [25]. Recently, several studies have reported carbapenem-resistant *Campylobacter* spp. isolates. In Tunisia, our previous work characterized the antimicrobial susceptibility and identified genes associated with resistance in *C. jejuni* and *C. coli* isolates from avian feces and related environments [25,26]. However, these isolates were not previously tested for carbapenem susceptibility, as carbapenems are not a first-line treatment for *Campylobacter* infections and the bacteria were presumed to be susceptible to this class of antibiotics. In light of emerging carbapenem resistance reported in other countries, we have now evaluated these isolates for carbapenem susceptibility and investigated the genetic determinants underlying this resistance.

High rates of resistance were observed toward several antibiotics including tetracycline, ciprofloxacin, erythromycin, and amoxicillin–clavulanic acid. Interestingly, despite full susceptibility to imipenem, resistance to meropenem and ertapenem were observed in 60.71% and 35.71% of *C. coli* isolates, respectively, and in 13.6% (for each antibiotic) of *C. jejuni* isolates. In addition, it is worth noting that all the 20 carbapenem-resistant *C. jejuni* isolates were co-resistant to meropenem and ertapenem; however, for *C. coli* isolates, 10.71%, 22.85%, and 66.42%, were resistant to ertapenem only, meropenem only, and to both ertapenem and meropenem, respectively. Zhuo et al. (2024) [14] have recently reported a case of recurrent multi-drug resistant *C. jejuni* bloodstream infections in a Bruton’s X-linked agammaglobulinemia patient receiving prolonged ertapenem therapy. The genetic basis of this resistance was associated with nonsynonymous mutations in the *porA* gene, which encodes the major outer membrane protein (MOMP). Mutations in *porA* may decrease the permeability of carbapenem on the basis of charge or size [14,15,27]. Similarly, Maurille et al. (2024) [16] have reported the occurrence of in vivo resistant *C. coli* isolated from a patient with Good’s syndrome under meropenem treatment. Resistance was mediated by *porA* point mutation and overexpression of *bla*_OXA-489_ under meropenem treatment. Taken together, mutations in PorA protein are the main mechanism of in vivo selection for carbapenem resistance in *Campylobacter* spp. [20,28]. However, a few studies have recently reported the emergence of carbapenemases enzymes in *Campylobacter*. The *bla*_OXA-185_ gene was recently reported in one ertapenem-resistant *C. jejuni* isolate recovered from chicken liver (healthy chicken from Catalonia, Spain) [28]. This gene has also been identified in 49 *C. jejuni* isolates from various sources (human, food, retail chicken, and environment) in USA and UK (https://www.ncbi.nlm.nih.gov/pathogens/isolates/#AMR_genotypes:blaOXA-185; accessed 10 August 2025).

In Gram-negative bacteria, the common carbapenemase enzymes belong to the Ambler class A (e.g., KPC types), class B (e.g., VIM, IMP, and NDM types), and the class D OXA β-lactamases [29,30]. NDM (New Delhi metallo-β-lactamase) and VIM (Verona integron-encoded metallo-β-lactamase) are metallo-β-lactamases that bind zinc ions to hydrolyze a broad spectrum of β-lactam antibiotics, including carbapenems. These enzymes are often encoded on mobile genetic elements, such as plasmids or integrons, which facilitate horizontal gene transfer. In contrast, OXA-48 is a class D β-lactamase (oxacillinase) that hydrolyzes carbapenems and penicillins, and is also frequently plasmid-borne, enabling its rapid dissemination [31]. Carbapenem-resistant Enterobacteriaceae have been detected in livestock, companion animals, and wildlife worldwide, although prevalence is generally low. Multiple carbapenemase genes, including NDM, VIM, KPC, OXA, and IMP, have been identified, mainly in *Escherichia* and *Klebsiella* species [32]. Global surveillance confirms the widespread dissemination of these carbapenemases beyond Enterobacterales. For instance, in *Pseudomonas aeruginosa* clinical isolates from Egypt, high rates of *bla*_VIM_ and *bla*_OXA-48_ have been reported in carbapenem-resistant strains [33]. Moreover, a study in Iran documented the co-existence of *bla*_NDM_, *bla*_VIM_, and other carbapenemase genes in multidrug-resistant *P. aeruginosa* isolates, along with intrinsic resistance mechanisms such as porin loss (OprD) and efflux-pump overexpression [34]. In *Acinetobacter baumannii*, molecular genotyping of clinical isolates in the Philippines revealed the presence of multiple carbapenemase genes, including *bla*_NDM_, *bla*_VIM_, and *bla*_OXA-48,_ often concurrently in the same strain [35].

In our collection, among the 20 carbapenem-resistant *C. jejuni* isolates, the carbapenemase-encoding genes *bla*_VIM_, *bla*_NDM_, and *bla*_OXA-48_ were detected in 15, 8, and 19 isolates, respectively. For *C. coli*, 53, 12, and 15 isolates carried the, *bla*_VIM_, *bla*_NDM_, and *bla*_OXA-48_ genes, respectively. To the best of our knowledge, this is the first report documenting the occurrence of carbapenemase-encoding genes in *Campylobacter* spp. isolates. This finding is alarming for two main reasons: (i) the potential for treatment failure when carbapenems are used as last-resort antibiotics, and (ii) the risk of horizontal transfer of these genes to commensal Gram-negative bacteria within the avian microbiome. In Tunisia, these genes have been frequently reported in clinical Gram-negative bacteria [36,37] but are rarely detected in isolates of animal origin [38], a situation likely related to the absence or scarcity of studies targeting carbapenem-resistant Gram-negative bacteria in animals. Most livestock-related research has instead focused on extended-spectrum β-lactamase (ESBL)-producing *Enterobacteriaceae* [39]. A recent study examining ESBL-producing *Escherichia coli* from calf feces, using cefotaxime-supplemented selective media, reported one ESBL-*E. coli* isolate co-harboring *bla*_OXA-48_ and *bla*_IMP_, and another isolate carrying only bla_IMP_ [40].Taken together, the detection of carbapenemase-encoding genes in *Campylobacter* of avian origin suggests the presence of a hidden reservoir of these genes and indicates that their prevalence in avian Gram-negative bacteria may be underestimated. Therefore, further studies on this issue are urgently needed.

The eleven *C. coli* isolates collected from the same farm and analyzed by MLST all belonged to the same sequence type, ST13450, which represents a newly identified ST. The clonal spread of MDR *C. coli* has been widely documented worldwide [41]. MLST analysis positioned the ST13450 isolates within an international genomic context, showing that this ST and its closely related clones are not confined to a specific region. Instead, they exhibit genetic relatedness to isolates reported across Europe, Africa, South America, and Asia. Such broad geographic distribution suggests cross-border dissemination, likely facilitated by international trade and human travel. Recent studies have shown that certain *Campylobacter* sequence types occur concurrently in both humans and poultry across different continents, reinforcing the hypothesis of globally circulating clones [42,43,44]. Consequently, the integration of ST13450 into this global clonal network highlights its dissemination potential and underscores the urgent need for coordinated worldwide genomic surveillance.

The analysis based on clinical outcome and source of isolation provides additional insight into the epidemiological behavior of ST13450. This sequence type was identified in both patients with gastroenteritis and asymptomatic carriers, demonstrating its capacity to cause disease while also circulating silently within the population. This dual behavior is particularly concerning because it enables hidden transmission pathways that are difficult to detect and control. Similar observations have been reported in Poland and South Korea, where *C. jejuni* and *C. coli* sequence types were found in both symptomatic cases and healthy carriers, reinforcing the major role of this genus in foodborne infections [45,46].

Another major finding is the close genetic relatedness between human isolates and those of animal origin (chicken and chicken meat), reinforcing the hypothesis of zoonotic transmission. This observation is consistent with previous reports from Tunisia, which have documented multidrug-resistant *Campylobacter* strains in poultry farms and carcasses carrying virulence genes and resistance determinants similar to those detected in human isolates [25,41]. The ability of ST13450 to persist and adapt across different hosts and environments highlights its emerging and versatile nature. This underscores the need for strengthened surveillance using a One Health approach that integrates human, animal, and food sectors to effectively monitor and control its spread.

## 4. Materials and Methods

### 4.1. Campylobacter Strain Collection

A total of 287 *Campylobacter* isolates, comprising 147 *C. jejuni* and 140 *C. coli*, were recovered from 590 broiler chicken fecal samples and 143 environmental samples collected from 23 poultry farms in three governorates in northeastern Tunisia (Ariana, Ben Arous, and Nabeul), which together account for 29% of national broiler production, between December 2016 and May 2018. All farms followed similar breeding practices, biosecurity/biosafety protocols, and bird numbers ranging from 2000 to 18,000 hens per house. Samples included cloacal swabs from chickens aged 15–40 days, as well as feces and water from the environment. The farms represented both intensive and semi-extensive production the farm systems, ensuring representative coverage of the Tunisian poultry sector. Chickens had not received antibiotics prior to sampling, and all collections were conducted using standard aseptic procedures.

### 4.2. Antimicrobial Susceptibility Tests

*Campylobacter* isolates were tested for susceptibility to antimicrobial drugs using the Kirby–Bauer disk diffusion method on Mueller–Hinton agar (Oxoid, Ltd., Basingstoke, UK) as recommended by the European Committee on Antimicrobial Susceptibility Testing [47]. All isolates were tested with the following antibiotics (Oxoid Ltd., Hampshire, UK): ampicillin (AMP, 10 μg), amoxicillin/clavulanic acid (AMC, 10/20 μg), gentamicin (GEN, 10 μg), streptomycin (SMN, 10 μg), kanamycin (K, 30 μg), nalidixic acid (NAL, 30 μg), ciprofoxacin (CIP, 5 μg), tetracycline (TET, 30 μg), erythromycin (ERY, 15 μg), azithromycin (AZM, 15 μg), linezolid (LIN, 10 μg), and chloramphenicol (CHL, 30 μg) [47].

### 4.3. Investigation of Carbapenemase Genes by PCR

Template DNAs for the PCR tests were extracted using the boiling method [26]. All isolates were screened for genes encoding most reported carbapenemases including *bla*_VIM_, *bla*_IMP_, *bla*_NDM_, and *bla*_OXA-48_ as previously described [48,49,50,51].

### 4.4. Statistical Analysis

Quantitative data, including prevalence of phenotypic resistance and resistance genes, were analyzed using the Chi-square test to compare differences between species (*C. jejuni* vs. *C. coli*) and between sample types (fecal vs. environmental). For all prevalence estimates, 95% confidence intervals (CIs) were calculated. A *p*-value < 0.05 was considered statistically significant, allowing robust assessment of differences in resistance profiles and gene distribution.

Associations between the presence of resistance genes (*bla*_VIM_, *bla*_NDM-1_, *bla*_OXA-48_) and carbapenem resistance phenotypes (ertapenem and/or meropenem) were evaluated using Chi-square (χ^2^) or Fisher’s exact tests, depending on data distribution. A binary logistic regression model was further applied to determine the independent effect of each gene on the likelihood of carbapenem resistance. For the logistic regression, resistance (1 = resistant to ertapenem and/or meropenem; 0 = susceptible) was used as the dependent variable, and the presence of the carbapenemase genes (*bla*_VIM_, *bla*_NDM-1_, *bla*_OXA-48_) as an independent predictor.

### 4.5. Multilocus Sequence Typing (MLST)

Eleven multidrug-resistant (MDR)/carbapenem-resistant *C. coli* isolates were selected to determine their clonal lineage by MLST. PCR amplicons identifying seven allele loci (*aspA*, *glnA*, *gltA*, *glyA*, *pgm*, *tkt*, and *uncA*) were obtained for each isolate by using the primers provided in PubMLSTdatabase (https://pubmlst.org/organisms/campylobacter-jejunicoli/primers, accessed on 25 November 2025). After sequencing of PCR products, ST profiles were assigned by submitting the sequences to the PubMLST database using the submission database [20].

### 4.6. Data Analysis

The heatmap analysis was conducted based on the antimicrobial susceptibility and the presence of genes encoding antibiotic resistance in the collected *Campylobacter* isolates. The results were visualized as heatmaps (Heatmaps 1 and 2) generated in Python (3.14.1) using the Seaborn (0.13.2) [52], Matplotlib (3.10.7) [53], and Pandas (2.3.3) [54] libraries, where phenotypic resistance values were represented by a color gradient ranging from green (low resistance) to red (high resistance). In parallel, the distribution of antimicrobial resistance genes *bla*_NDM-1_, *bla*_IMP_, *tet(O), cmeB*, *bla*_OXA-61_, *bla*_OXA-48_, and *bla*_VIM_, was compared between *C. jejuni* and *C. coli* (ST13450) and visualized as a clustered heatmap (Heatmap 2), in which color intensity reflects gene prevalence, and hierarchical clustering was applied to explore genetic similarity patterns.

Phylogenetic relationships of the eleven isolates studied by MLST were reconstructed with the GrapTree tool (version 1.1.0, available within BIGSdb), which generates minimum spanning trees based on allelic distances. In the resulting networks, each node corresponds to a sequence type (ST), node size is proportional to the number of isolates, edges indicate the number of allelic differences, and node colors represent either the country of origin or the clinical/animal source of isolation. This approach enabled the visualization of genetic relatedness among isolates and provided insights into their geographical distribution and epidemiological context.

## 5. Conclusions

The detection of carbapenemase-producing *Campylobacter* in poultry and their surrounding environments underscores the critical need for comprehensive surveillance programs. The presence of genes encoding VIM, OXA-48, and NDM enzymes, which are already widespread in clinically important human pathogens such as Enterobacterales, *P. aeruginosa*, and *A. baumannii*, raises concern about the potential dissemination of these resistance determinants from food animals to humans. Systematic monitoring of antimicrobial resistance in both animal and environmental samples can identify emerging resistance patterns and high-risk areas, enabling timely and targeted interventions. Promoting antimicrobial stewardship in poultry production, including rational antibiotic use, adoption of alternative therapies, and enhanced biosecurity, can help reduce selective pressures that drive resistance. From a regulatory perspective, these findings highlight the importance of farm-level guidelines, One Health-oriented policies, and risk-based strategies to limit the spread of carbapenem-resistant bacteria through the food chain. Collectively, these measures are essential for safeguarding public health and preserving the efficacy of critically important antibiotics. However, a key limitation of this study is the lack of data on *Campylobacter* from humans in Tunisia, which prevents direct assessment of zoonotic transmission or the establishment of clear links between poultry and human infections. Future investigations integrating human, animal, and environmental surveillance would be essential to clarify potential transmission pathways and to guide targeted public health interventions. Collectively, these measures are essential for safeguarding public health and preserving the efficacy of critically important antibiotics.

## Figures and Tables

**Table 1:** Frequencies of detected genes in the C. coli and *C. jejuni* isolates.

Species	Antibiotic Resistance Genes (%)							MLST **
	*cmeB*	*tet*(O)	*bla*_OXA-61_	*bla*_NDM-1_ * (*n* = 20, 6.9%)	*bla*_OXA-48_ * (*n* = 34, 13.9%)	*bla*_VIM_ * (*n* = 68, 23.6%)	*bla*_IMP_	
*C. jejuni* (n = 147)	80%	100%	81%	40% (8/20) (Er/Mer)	95% (19/20) (Er/Mer)	37.5% (15/40) (Er/Mer)	0%	-
*C. coli* (n = 140)	100%	80%	93%	12.9% (12/93) (Er/Mer)	16.12% (15/93) (Er/Mer)	39.25% (53/135) (5 Er + 18 Mer 30 Er/Mero)	0%	ST13450

* Phenotype of resistance to ertapenem (Er) and meropenem (Mer). For carbapenemase genes the percentages were determined according to the number of isolates resistant to carbapenems. ** The 11 *C. coli* isolates were resistant to ertapenem (Er) and meropenem (Mer).

## Data Availability

The statistical data used to support the findings of this study are available from the corresponding author upon request.
